# The learning experiences of mentees and mentors in a nursing school's mentoring programme

**DOI:** 10.4102/curationis.v38i1.1145

**Published:** 2015-03-24

**Authors:** Annemarie Joubert, Johanna de Villiers

**Affiliations:** 1 School of Nursing, University of the Free State, South Africa

## Abstract

**Background:**

A School of Nursing supports third-year undergraduate students (mentees) by means of a mentoring programme in which critical-care nursing students (mentors) are involved. However, the programme designers needed to find out what gaps were evident in the programme.

**Objectives:**

The objectives of the study were to explore and describe the learning experiences of the mentees and mentors and to obtain recommendations for improving the programme.

**Method:**

An action-research method was used to develop and to refine the student-mentoring programme and to identify student needs. However, for the purposes of this article a descriptive design was selected and data were gathered by means of a nominal-group technique. Fourteen mentees and five mentors participated in the research.

**Results:**

The findings indicated that attention should be paid to the allocation and orientation of both mentors and mentees. Amongst the positive experiences was the fact that the mentees were reassured by the mentor's presence and that a relationship of trust developed between them. In consequence, the mentees developed critical thinking skills, were able to apply their knowledge and improved their ability to integrate theory and practice. Not only did the mentees gain respect for the mentors’ knowledge and competence, but they also lauded the mentoring programme as a memorable and vital experience.

**Conclusion:**

The findings indicated that several changes would be needed to improve the structure of the mentoring programme before a new group of mentees could be placed in critical-care units.

## Introduction

The School of Nursing in the Free State Province of South Africa has a student-mentoring programme (SMP) which addresses the social, academic and clinical needs of undergraduate nursing students through the implementation of different strategies. These strategies include pairing first-year students with third-year students in order to support them during their first placement in the clinical environment, or by using senior nursing students with exceptionally high marks in anatomy, chemistry or microbiology to support students who struggle with these subjects. This article focused on the support rendered by post-basic nursing students enrolled in the critical-care nursing programme to third-year undergraduate nursing students placed in the critical-care units so as to assist them in meeting the module outcomes. In the post-basic critical-care programme, students are required to be involved in the clinical teaching of third-year undergraduate students. Prior to their placement in the different critical-care units, the third-year students (mentees) and critical-care nursing students (mentors) are briefed on their different roles and responsibilities. A structured guideline stating, for example, the outcomes that should be met by both mentors and mentees is also provided. Twelve mentors were available to facilitate 55 mentees and each mentor was scheduled to spend at least 48 hours with each mentee during the month the mentee was placed in the critical-care unit.

### Background

The utilisation of mentor support in the academic and clinical environments is described in the research literature. Mentor support was seen as being central to a Clinical Practice and Placement Support Unit (CPPSU) developed at the University of Dundee, Scotland. Not only did mentors provide direct support to enhance the development of clinical skills, but they also ‘engage the student in critical thinking, reflection on practice and an exploration of alternative strategies to care’ (Burns & Paterson 2004). Furthermore, the results of a randomised controlled trial of a graduate-to-graduate student mentoring programme confirmed its effectiveness with regard to lowering of anxiety, improvement of academic performance and satisfaction with nursing as a career in the experimental group (Kim *et al.* 2013:e46).

The importance of student support in the development of competence and critical thinking or higher-order skills is also argued by Letizia and Jennrich (cited in Udlis 2006:21) and Wilson *et al.* (2011:154). Mentorship, as seen by Letizia and Jennrich, ‘ought to produce more clinically competent, critically thinking, and professionally prepared graduate nurses’.

### Problem statement

In 2009, the facilitators of the third-year undergraduate and the post-basic critical-care nursing students decided to link the extensive support that should be rendered to third-year nursing students (mentees) during their placement in the critical-care clinical environment with the module requirements for clinical teaching of the post-basic critical-care nursing students.

The decision to provide extensive support to the third-year nursing students was based on research findings that students experienced a ‘lack of teaching and learning support’ and ‘poor theory-practice integration’ (Mabuda, Potgieter & Alberts 2008:19). Furthermore, exposure to new clinical learning environments caused additional emotional pressure. This increased anxiety and tension in student nurses, preventing the students from making the best possible use of the clinical experience (Bunce 2002:24; Carlson, Kotzé & Van Rooyen 2003:30). According to Anderson (2011:52), experienced and knowledgeable clinical staff promote clinical learning of students by creating an effective learning environment.

The authors of the current article agree with Burns and Paterson (2004) and Hattingh, Coetzee and Schreuder (2005) in that nursing students should be exposed to a constantly changing and challenging teaching and learning environment. The authors also acknowledge the fact that the decisions regarding the support of undergraduate students should be based on consistent feedback and input from mentees and mentors. Because, for the first time, third-year students were mentored by post-basic critical-care nursing students, the researchers decided to evaluate the experiences of both groups.

## Research questions

What are the experiences of mentees and mentors in a School of Nursing's mentoring programme?

What recommendations do mentees and mentors have to improve a School of Nursing's mentoring programme?

### Purpose and paradigm

The dual purpose of the research on which this article is based was: (1) to explore and describe the learning experiences of third-year undergraduate and post-basic critical-care nursing students who were involved in the School of Nursing's student mentoring programme in 2010; and (2) to make a number of recommendations based on the feedback received.

Ontology is referred to as ‘a theory of being, how we see ourselves’ (McNiff & Whitehead 2009:8). The researchers regarded the mentees as junior colleagues who needed to be supported to become professional practitioners. Mentors, although considered to be at a more advanced level of competence, could still benefit through their involvement in the process of mentoring.

### Concept clarification

Mentoring is defined by Hawkins and Fontenot (2010) (quoted in Botma, Hurter & Kotze 2012:808) as being a relationship between two people that is interdependent, cultivating and trusting. The experienced and competent practitioner guides and supervises the mentee or less experienced practitioner. Being a role model is an important characteristic of the mentor. The mentoring programme refers to a year programme that was designed by the School of Nursing to support undergraduate nursing students academically and during their placements in different clinical environments. The mentors are post-basic critical-care nursing students who act as academic and social supporters but, more specifically, provide advanced support to third-year undergraduate nursing students or mentees in the critical-care work-based or clinical environment. The mentees are third-year undergraduate nursing students who received clinical support from the mentors. Learning experiences are defined as ‘instructional strategies’ designed by faculty and implemented with the aim of providing ‘specific kinds of student learning experiences’ (Billings & Halstead 2005:154). In this article, ‘learning experiences’ refers to the mentees’ reflection on or experience of the support rendered by mentors as an instructional strategy.

## Research methods and design

A qualitative descriptive design was used, based on the attributes described by (Botma *et al.* 2010:182). The reporting of data in a ‘literary style rich with participant commentaries’ was the departure point to describe the learning experiences of mentees and mentors in the SMP. Data collection was done by using the nominal-group technique (Potter, Gordon & Hamer 2004:126).

The nominal-group technique included 67 members and consisted of specific persons interested in or involved with a particular section of the problem under investigation, headed up by a facilitator (Botma *et al.* 2010:251–253; Center for Rural Studies 1998; De Vos *et al*. 2011:503; Mycoted 2007). An internal facilitator with experience in facilitating nominal groups was used, because of the limited availability of external facilitators and the requirement that the facilitator had to be able to capture the feedback by using MindManager Software from Mindjet (http://www.mindjet.com/mindmanagerv.10.0.4952012). Through this technique, creative ideas were generated, the results interpreted and the available time used efficiently (Jones 2004:23–24).

Creswell (2003:105) recommends that explanatory questions, as well as a main and a follow-up question be used. Consequently, instead of research questions, the following main and follow-up tasks or instructions were given to the participants: ‘Write down your experience of the SoNs mentoring programme’, and ‘Write down recommendations on how the SoNs student mentor programme could be improved’.

## Population

A population is defined by Polit and Beck (2012:738) as being a group of individuals or objects that shares the same characteristics, or a collection of cases that fits the purpose of a study.

In this study, the population was 55 third-year undergraduate (mentees) and 12 post-basic nursing critical-care students (mentors) registered at a University.

### Unit of analysis or sample and sample size

The unit of analysis is the type of unit, for example, individuals, organisations, or groups that researchers use when measuring variables (Botma *et al*. 2010:51; De Vos *et al*. 2011:93; Green & Thorogood 2009:138). To identify these units, researchers should be guided by the topic, problem and purpose of the study (De Vos *et al*. 2011:93).

Participants were invited purposefully, based on their ability to answer the research question and therefore generating a deeper understanding of the mentor-mentee issues in this article. Eventually 14 (14/55) third-year mentees and 5 (5/12) post-basic critical-care nursing mentors who were willing to participate were included in the nominal-group discussions.

### Nominal-Group Technique: Preparation and execution

Because a nominal-group technique (NGT) is a ‘structured method that encourages contributions from everyone’ (Tague 2005:364–365), the following measures were taken in the study. The 14 mentees and 5 mentors participated in separate nominal groups. The participants could also indicate whether they preferred an Afrikaans or English group (Van de Ven & Delbecq 1972). An informal environment was created by careful selection of a large enough venue. Participants were seated in a horseshoe or U-shaped format (Dobbie *et al.* 2004:403; Dunham 1998; Potter *et al.* 2004:126). The feedback from the participants was captured electronically by the facilitator using MindManager, a software program designed to visualise information and to refine it into results.

A four-step process was used to conduct the nominal-group interviews: generating; capturing or sharing of data; discussing and clarification; and voting on categories to establish priorities (Botma *et al*. 2010:251–252; Department of Health and Human Services 2006). An experienced facilitator emphasised the importance of each member's contribution, then introduced and clarified the instructions. Team members were given time to think, reflect on and write down their responses. The cycle was repeated until all the responses had been obtained.

## Data treatment and analysis

Data treatment and analysis were completed during the nominal-group interview discussion and voting process. The data were analysed and checked by the researchers according to the multiple-group analysis of Van Breda (2005). Suggestions by Potter *et al.* (2004:127), namely the direct involvement of the participants in the data-collection and analysis process, ensured objectivity on the part of the researchers.

McNiff and Whitehead (2009:327) mention that all research should meet the requirements for rigour. The same authors quote Stringer (1999) who states that rigour assured by utilising the criteria of trustworthiness, namely credibility, transferability, dependability, and confirmability, as indicated by Lincoln and Guba (1985:289).

The researchers adhered to the criteria described by Lincoln and Guba (1985:289) and followed the methods of data collection, analysis and interpretation described in their research proposal. Furthermore, the researchers explained to the participants what their roles and responsibilities during the process of data collection will be. The participants confirmed that the information discovered during the NGTs was a true reflection of their experiences. The pledge of confidentiality made to the participants was kept. The researchers had no hidden agenda and ensured that the interests of the subjects, as well as their input into the inquiry, were honoured.

## Ethical issues

An ethics number issued by the Ethics Committee Faculty of Health Sciences was obtained by the researchers before the implementation of the research. The proposed guidelines – for example, respect for the participants’ right to information regarding the research and the outcomes thereof – were adhered to (Bouma & Ling 2004:192; Brink 1999:39). The participants provided written consent and had the right to discontinue their participation in the study without being discriminated against (Cherry 2000:23). Data were kept in a safe and were available for enquiries (Burns & Grove 2009:196–197). To ensure confidentiality, no personal information is mentioned in the article. Furthermore, the researchers strived to maintain the trust of the participants; consequently, an objective and honest approach in reporting findings was a priority (Burns & Grove 2005:195).

## Results

This section describes the results regarding the learning experiences of the mentors and mentees according to the categories identified by each group. A consensus method was used to determine the priorities, with all five mentors voting.

### Learning experiences of mentors (critical-care students, post-basic programme)

The learning experiences of the mentors were evident in the five categories that they identified. These categories included: allocation; correlation and application of theory in practice; mentee attitude; learning experience; and reassurance and trust.

#### Allocation

The statements made by mentors indicated that performing the role of a mentor in a critical-care unit environment was considered difficult under certain circumstances. For example, striking a balance between overloading the mentees with work and underexposing them to important nursing issues was a challenge. On the one hand, the mentors found it overwhelming to look after two students on a busy day. On the other hand, they mentioned that the time they (the mentees) were exposed to the mentor was too short for the amount of information that needed to be disseminated. The mentors agreed that for them, ‘it was better to mentor students for a shift rather than an hour’. From the perspective of mentors working in critical-care units, the number of mentees that was allocated could make it difficult to assist them all.

#### Correlation and application of theory in practice

The research findings clearly indicated that mentoring facilitated the participating mentees’ ability to correlate and apply theory in a critical-care environment. The mentors confirmed that the mentees’ demonstrated skills such as critical thinking and the application of knowledge. The mentors were also convinced that they were able to provide the mentees with the opportunity to experience evidence-based practice.

#### Mentee attitude

The mentors valued certain characteristics in their mentees and voiced their appreciation of the mentees’ attitudes toward their clinical responsibilities, stating, ‘[*t*]he mentees were always willing to help to make the flow of the unit easier, for example to address basic needs’. Unfortunately, instances where mentees did not meet the expectations of mentors were also experienced. According to the mentors, some students did not show enough interest and came unprepared to the clinical areas.

#### Learning experience

The mentors stated that mentoring required much more than just the willingness to participate in a mentoring programme. Being a mentor made them reflect on their own competencies and, according to them, forced them to go back to their books to read up again.

#### Reassurance and trust

The mentors agreed that the mentees appreciated their presence, stating, ‘[*t*]he mentees felt reassured due to the fact that the mentors were there’. However, when the mentors had other responsibilities, such as going on doctors’ rounds, they had to trust the mentees to provide the necessary care.

### Learning experiences of mentees (students in undergraduate programme)

The same task with regard to their learning experiences was given to the mentees. The five issues that received priority were: availability; knowledge and competency; mentor attitude; mentor support; and theory and practice integration. A consensus method was used to determine the priorities, with all 14 mentees voting.

#### Availability

The mentees perceived the limited availability of the mentors as being problematic. In some cases, the mentors were available only very late in the year or not available at all. Mentees mentioned that they were placed with the mentors in October for the first time. Furthermore, the mentees stated that the registered nurse in the unit had to explain the work because the mentors had not been there. Achieving the stated outcomes seemed to be an important issue for the mentees and they were concerned about the fact that the mentors seemed to be unaware of how important the outcomes were, stating, ‘[*t*]he mentor did not know how important the outcomes were and did not organise support from other critical-care unit staff [*referring to permanent staff members*] to provide help in order to obtain outcomes’. Despite the mentees’ concerns, being mentored was still described as good experience, despite the fact that little time (supernumerary status of mentee not always adhered to by nurse-in-charge of critical care) was spent with the mentors.

#### Knowledge and competency

One positive aspect of the mentoring programme was the mentees’ regard for the mentors’ capabilities (theoretical knowledge and clinical skills). They described the mentors as being knowledgeable and competent.

#### Attitude of mentor and support rendered

The characteristics of mentors and their supportive role influenced the way the mentees experienced their exposure to mentors in the mentoring programme. The mentors were described by the mentees as being ‘enthusiastic and helpful’ and it seemed that the mentors did meet some of the mentees’ expectations. The mentees stated that they found the mentors to be positive and appreciated the fact that mentors participated voluntarily in the programme because they wanted to teach the mentees. The mentees also acknowledged and appreciated the support that was rendered by the mentors, stating that the mentors were there to assist them.

#### Theory and practice integration

The participants experienced mentoring as being positive with regard to the integration of theory and practice. The mentees confirmed that theory and practice integration had taken place and that they subsequently understood more about patients and patient care in all its facets in critical-care units.

#### Vital role

The educators involved in the design and implementation of this mentoring programme were serious about the outcome. However, even though the mentees described the programme as an ‘awesome workout for mentor and mentee’ and that ‘the mentor programme plays a vital role in training the students’, the following recommendations by mentors and mentees, for example, orientation, allocation and selection of mentors, will have to be addressed.

### Recommendations by mentors and mentees on how to improve the existing programme

Despite previous efforts to upgrade and facilitate the student mentoring programme before the study was completed, the mentors and mentees still made the following recommendations to address their needs.

#### Orientation

It is stressed that the orientation of mentors and mentees regarding their roles, responsibilities and the outcomes of the programme could be underestimated. More attention should be paid to the timing of orientation, the expected outcomes stated for mentees and the introduction of mentors to the mentees. Mentees requested that critical-care unit mentors be oriented early in the year and that mentees should be updated on what is expected of them, for example, regarding their scope and clinical outcomes. Furthermore, enough time should be allocated for mentors and mentees to bond and for mentees to be briefed on important matters. Mentors must meet beforehand to discuss what the mentees will learn and to explain to mentees what is expected.

#### Allocation

From the research, it was evident that better allocation of mentors and mentees with regard to the number, duration and time would be needed. The mentors were willing to accompany more than one student. Mentors indicated that they preferred to accompany a maximum of three students per month and per unit. However, the mentees were of a different opinion and expressed the opinion that ‘two mentors for each mentee in case one is not available’ and that the number of mentees per mentor should be limited, working on a one-to-one basis only. The mentees were also less satisfied with the duration of placements and their recommendation that they should be allocated for one week of full-time mentoring would definitely be considered. The time when placements would be done was critical and an important recommendation was that mentees should be placed earlier.

It was further evident from the research that much more effort had to be made when dealing with the day-to-day critical-care unit staff. The tendency to use mentees and mentors for daily activities did create problems with regard to the outcome of the programme. It was reported that the responsibility of the mentoring programme coordinator is to inform the matrons responsible for training. The mentees felt that, ‘[*m*]atrons must be made aware that you are in a mentoring programme and allocate you to the mentor’.

#### Multidisciplinary approach

Facilitation and coordination of the involvement of a multidisciplinary team in the training of nurses are difficult. However, as suggested by the mentors, the possibility of involving other members of a multidisciplinary team in a SMP should be investigated. According to the mentors, ‘[*i*]t will be nice if other disciplines also become involved in mentoring’.

#### Opportunities and outcomes

The mentees considered the learning opportunities and identified clinical outcomes as being important. They requested that learning opportunities should be practical; for example, mentors should explain the difference and interaction between diagnostic results and laboratory results. An increased number of opportunities (learning opportunities related to caring for critical-care patients) were also recommended. The fact that the mentors had to be aware of the importance of the objectives was also voiced.

#### Selection

The mentees suggested that more attention be paid to the selection process. The group confirmed that ‘[*m*]entoring should be voluntary – some nurses do not want to be mentors’. The mentees requested that the selection of mentors be done with care.

## Discussion

Both mentors and mentees mentioned both positive and negative experiences and were more than willing to make recommendations to improve the quality of the existing programme.

From the research findings, it is evident that careful consideration should be given to the preparation and orientation, not only of mentors and mentees, but also of unit staff and unit managers. Wilson, Sanner and McAllister (2010:144) have used focus-group interviews to assess the perceptions of faculty mentors and student mentees regarding a preparation and orientation programme. The results of the focus-group interviews showed that the deliberate and extensive preparation and orientation to become mentors contributed to the uniqueness of the programme (Wilson *et al.* 2010:144).

The mentees who participated in our study definitely viewed as important the orientation or guidance that critical-care unit mentors received before they were allocated to third-year mentees. Giordana and Wedin (2010:395), Dennison (2010:341) and Jackson *et al.* (2003:333) confirm that orientation regarding different roles and responsibilities is important. Not only is the coordinator responsible for matching mentors and mentees, but the placement and responsibilities should be done in consultation with the mentors (Metcalfe 2010).

Allocations, specifically with regard to the duration or number of hours that mentees should spend with the mentor, were also addressed by other researchers. Taylor and Neimeyer’s (2009:260, 262) research at the University of Florida has focused on graduate student mentoring within clinical, counselling and experimental psychology programmes. The final analysis of their research regarding the number of hours mentees spent with mentors correlates positively with socio-emotional support. According to Taylor and Neimeyer (2009:260, 262), as protégés gain autonomy and self-sufficiency, they become ‘more independent’ and less reliant on their mentors.

The outcomes stated in a mentoring programme are crucial. For example, when it comes to the development of critical thinking and the application of knowledge, the value of mentoring cannot be ignored. Dennison (2010:340) lists assistance in the development of skills and critical thinking as one of the primary functions of mentors at a southern Ontario university. According to Ferguson (2010), the new nurses appreciate senior students who share knowledge.

Careful selection of mentors was requested by mentees in the current study. During the selection process, the characteristics of the mentees, their ability to provide support and to act as role models, should be considered. Naturally, mentors should also be selected based on their competencies or skills.

The characteristics and supportive role of mentors, as mentioned by mentees in the current study, were researched extensively. Dennison (2010:340), Ferguson (2010) and Metcalfe (2010) state that a characteristic of peer mentors is to be supportive and to create an open and comfortable learning environment for mentees. Giordana and Wedin (2010) have used an exploratory research method to investigate peer mentoring for multiple levels of nursing students. Mentees did not feel ‘intimidated’ or ‘vulnerable’ in the presence of the mentor. One mentee compared being with the mentor as having a ‘security blanket’.

Wilson *et al.* (2010:144) have used focus-group interviews to evaluate the perceptions of mentors and mentees regarding a mentoring programme. Role modelling is one of the themes that they have identified. Mentors believe that they are able to display the characteristics and the type of activities required by nurses to mentees. Mentees see mentors as experienced, able to share their knowledge and able to foster leadership skills in others (Jackson *et al.* 2003:329; Metcalfe 2010). Tobin (2004:116) writes about ‘picking a mentor’ and states that enthusiasm is the most important quality to search for.

The above views are reflected in several research reports (Byrne & Keefe 2002:395; Jackson *et al.* 2003; Metcalfe 2010; Taylor & Neimeyer 2009:260, 262; Tobin 2004:116) that address the roles and characteristics of mentors.

Mentors also benefit from being involved in a SMP. In addressing the needs of mentees, mentors are also challenged regarding their own knowledge and skills and encouraged to make time to revisit the available resources. The finding that mentors have the need to update their knowledge is confirmed by Dennison (2010:341). Peer mentoring at a southern Ontario university challenged mentors regarding the scope of questions posed by mentees. The mentors also noticed how much they had learned and that they had learning needs of their own.

The value of being mentored is also confirmed in other studies. Dennison (2010:340) describes mentoring as being a ‘valuable educational strategy’ that correlates positively with the development of trust and fostering a collegial relationship. Tobin (2004:115) discusses trust in the mentor–mentee relationship and argues that the mentor ‘serves a as confidante’ and that the two-way relationship is ‘based on trust’. Both authors’ views are supported by Wilson *et al.* (2010:145–147).

### Limitations

The disadvantages that were considered were the limited number of topics and issues that can be covered; the limited opportunity for participants to think about the issues; and the lack of anonymity, which may limit participants’ willingness to express their views (Jones 2004:23–24).

## Conclusion

Despite the fact that the SMP still requires attention with regard to logistics, both mentees and mentors seemed to have benefitted from being involved in the programme. The insights gained, for example, with regard to the number of mentees allocated to a mentor, the number of hours mentees should spend with mentors, orientation concerning the different roles and the request for a multidisciplinary approach could be used to improve the quality of support rendered to both mentees and mentors. It is hoped that, in future, students’ needs and throughput will also be addressed in the process.

## References

[CIT0001] Anderson L., 2011, ‘A learning resource for developing effective mentorship in practice’, *Nursing Standard* 25(51), 48–56. 10.7748/ns2011.08.25.51.48.c8661 21922743

[CIT0002] Billings D.M. & Halstead J.A., 2005, *Teaching in nursing: A guide for faculty*, 3rd edn., Saunders Elsevier, Cape Town.

[CIT0003] Botma Y., Greeff M., Mulaudzi F.M. & Wright S.C.D., 2010, *Research in health sciences*, Heinemann, Cape Town.

[CIT0004] Botma Y., Hurter S. & Kotze R., 2012, ‘Responsibilities of nursing schools with regard to peer mentoring’, *Nurse Education Today* 33(8), 808–813. 10.1016/j.nedt.2012.02.021 22464630

[CIT0005] Bouma G.D. & Ling R., 2004, *The research process*, 5th edn., Oxford, New York.

[CIT0006] Brink H.I., 1999, *Fundamentals of research methodology for health care professionals*, Juta Academic, Cape Town.

[CIT0007] Bunce C., 2002, ‘Placement blues’, *Nursing Times* 98(17), 24–26. 12008256

[CIT0008] Burns N. & Grove S.K., 2005, *The practice of nursing research: Conduct, critique and utilization*, 5th edn., Elsevier, St Louis, MO.

[CIT0009] Burns N. & Grove S.K., 2009, *The practice of nursing research: Conduct, critique and utilization*, 6th edn., Elsevier, St Louis, MO.

[CIT0010] Burns I. & Paterson I.M., 2004, ‘Clinical practice and placement support: Supporting learning in practice’, *Nurse Education in Practice* 5(1), 3–9. 10.1016/j.nepr.2004.02.001 19038173

[CIT0011] Byrne M.W. & Keefe M.R., 2002, ‘Building research competence in nursing through mentoring’, *Journal of Nursing Scholarship* 34(4), 391–396. 10.1111/j.1547-5069.2002.00391.x 12501744

[CIT0012] Carlson S., Kotzé W.J. & Van Rooyen D., 2003, ‘Accompaniment needs of first year nursing students in the clinical learning environment’, *Curationis August*, 30–39. 10.4102/curationis.v26i2.778 14596131

[CIT0013] Center for Rural Studies, 1998, *Guidelines for using the nominal group technique*, viewed 03 January 2015, from https://www.uvm.edu/crs/resources/nerl/group/a/meet/Exercise7/b.html

[CIT0014] Cherry A.L., 2000, *A research primer for the helping professions: methods, statistics, and writing*, Wadsworth, Belmont.

[CIT0015] Creswell J.W., 2003, *Research design: Qualitative, quantitative, and mixed methods approaches*, 2nd edn., Sage, California.

[CIT0016] Dennison S., 2010, ‘Peer mentoring: untapped potential’, *Journal of Nursing Education* 49(6), 340–342. 10.3928/01484834-20100217-04 20210287

[CIT0017] Department of Health and Human Services, 2006, *Gaining consensus among stakeholders through the nominal group technique*, Evaluation Briefs, No. 7, viewed 20 July 2009, from http://www.cdc.gov/healthyyouth/evaluation/index.htm

[CIT0018] De Vos A.S., Strydom H., Fouché C.B. & Delport C.L.S., 2011, *Research at grass roots: A primer for the social science and human service professions*, 4th edn., Van Schaik Publishers, Pretoria.

[CIT0019] Dobbie A., Rhodes M., Tysinger J.M. & Freeman J., 2004, ‘Using a modified nominal group technique as a curriculum evaluation tool’, *Family Medicine* 36(6), 402–406. 15181551

[CIT0020] Dunham R.B., 1998, Nominal group technique: A user's guide, viewed 03 January 2015, from http://www.sswm.info/sites/default/files/reference_attachments/DUNHAM%201998%20Nominal%20Group%20Technique%20-%20A%20Users%27%20Guide.pdf

[CIT0021] Ferguson L.M., 2010, ‘From the perspective of new nurses: What do effective mentors look like in practice?’, *Nurse Education in Practice* 11(2), 119–123. 10.1016/j.nepr.2010.11.003 21159558

[CIT0022] Giordana S. & Wedin B., 2010, ‘Peer mentoring for multiple levels of nursing students’, *Nursing Education Perspectives* 31(6), 394–396. 21280450

[CIT0023] Green J. & Thorogood N., 2009, *Qualitative methods for health research*, 2nd edn., Sage Publications Ltd., London.

[CIT0024] Hattingh M., Coetzee M. & Schreuder D., 2005, ‘Implementing and sustaining mentoring programmes: A review of the application of best practices in the South African organisational context’, *SA Journal of Human Resource Management* 3(3), 40–48. 10.4102/sajhrm.v3i3.73

[CIT0025] Jackson V.A., Palepu A., Szalacha L., Caswell C., Carr P.L. & Inui T. 2003, ‘“Having the right chemistry”: A qualitative study of mentoring in academic medicine’, *Academic Medicine* 78(3), 328–334. 10.1097/00001888-200303000-00020 12634219

[CIT0026] Jones S.C., 2004, ‘Using the nominal group technique to select the most appropriate topics for postgraduate research students’ seminars’, *Journal of University Teaching & Learning Practice* 1(1), 20–34.

[CIT0027] Kim S.C., Oliveri D., Riingen M., Taylor B. & Rankin L., 2013, ‘Randomized controlled trial of graduate-to-undergraduate student mentoring program’, *Journal of Professional Nursing* 29(6), e43–e49. 10.1016/j.profnurs.2013.04.003 24267940

[CIT0028] Lincoln Y.S. & Guba E.G., 1985, *Naturalistic inquiry*, Sage, Newbury Park, CA.

[CIT0029] Mabuda B.T., Potgieter E. & Alberts U.U., 2008, ‘Student nurses’ experiences during clinical practice in the Limpopo Province’, *Curationis* 31(1), 19–27. 10.4102/curationis.v31i1.901 18592945

[CIT0030] McNiff J. & Whitehead J., 2009, *All you need to know about Action Research*, Sage Publications Ltd., London.

[CIT0031] Metcalfe S.E., 2010, ‘Educational innovation: Collaborative mentoring for future nursing leaders’, *Creative Nursing* 16(4), 167–170. 10.1891/1078-4535.16.4.167 21140869

[CIT0032] Mycoted, 2007, *Nominal group technique*, viewed 04 March 2007, from http://www.mycoted.com/Nominal_Group_Techique

[CIT0033] Polit D.F. & Beck C.T., 2012, *Nursing research: Generating and assessing evidence for nursing practice*, 9th edn., Wolters Kluwer/Lippincott Williams & Wilkins, New York, NY.

[CIT0034] Potter M., Gordon S. & Hamer P., 2004, ‘The nominal group technique: A useful consensus methodology in physiotherapy research’, *New Zealand Journal of Physiotherapy* 32(3), 126–130.

[CIT0035] Tague N.R., 2005, *The quality toolbox*, 2nd edn., American Society for Quality, Quality Press, Milwaukee, WI.

[CIT0036] Taylor J.M. & Neimeyer G.J., 2009, ‘Graduate school mentoring in clinical, counselling, and experimental academic training programs: an exploratory study’, *Counselling Psychology Quarterly* 22(2), 257–266. 10.1080/09515070903157289

[CIT0037] Tobin M.J., 2004, ‘Mentoring: Seven roles and some specifics’, *American Journal of Respiratory and Critical Care Medicine* 170(2), 114–117. 10.1164/rccm.2405004 15242852

[CIT0038] Udlis K.A., 2008, ‘Preceptorship in undergraduate nursing education: An integrative review’, *Journal of Nursing Education* 47(1), 20–29. 10.3928/01484834-20080101-09 18232611

[CIT0039] Van Breda A.D., 2005, ‘Steps to analysing multiple-group NGT data’, *The Social Work Practitioner-Researcher* 17(1), 1–14.

[CIT0040] Van De Ven A.H. & Delbecq A.L., 1972, ‘The nominal group as a research instrument for exploratory health studies’, *American Journal of Public Health* 62(3), 337–342. 10.2105/AJPH.62.3.337 5011164 PMC1530096

[CIT0041] Wilson A.H., Sanner S. & McAllister L.E., 2010, ‘An evaluation study of a mentoring program to increase the diversity of the nursing workforce’, *Journal of Cultural Diversity* 17(4), 144–150. 22303649

[CIT0042] Wilson Z.S., Holmes L., DeGravelles K., Sylvain M.R., Batiste L., Johnson M. et al., 2011, ‘Hierarchical mentoring: A transformative strategy for improving diversity and retention in undergraduate STEM disciplines’, *Journal of Science Education and Technology* 21(1), 148–156. 10.1007/s10956-011-9292-5

